# Review of the cellulose acetate peel method and the physical and digital curation of coal balls

**DOI:** 10.1002/aps3.11556

**Published:** 2023-11-29

**Authors:** Scott R. Lakeram, Scott Elrick, Surangi W. Punyasena

**Affiliations:** ^1^ Department of Plant Biology University of Illinois Urbana‐Champaign 505 S. Goodwin Ave. Urbana Illinois 61801 USA; ^2^ Illinois State Geological Survey 615 E. Peabody Drive Champaign Illinois 61820 USA

**Keywords:** Carboniferous, coal ball, imaging, paleobotany, peel method

## Abstract

Coal balls, in which fossil plants are preserved in permineralized peat deposits, have widely been described from coal deposits representing the tropical forest of the Carboniferous. Coal ball preparation techniques have evolved over the past century, with the cellulose acetate peel method becoming the standard in the 1950s. While coal ball research is not as active as it has been in the past, large collections of coal balls and their respective peels still form a large part of many museum and university collections. This contribution aims to review coal ball preparation methods, curation, and the digital archiving of peels to create a cohesive guide for researchers working with coal balls and other petrified plant material. The physical and digital curation of cellulose acetate peels and other types of coal ball specimens is critical for long‐term preservation and accessibility. Physical curation involves embedding coal balls in media to slow pyrite deterioration. Digital curation creates high‐resolution scans of peels, which can be shared and accessed online. Cellulose acetate peels and their digital curation are a valuable and accessible technique for the analysis of coal balls, and physical and digital curation ensures long‐term preservation.

Coal balls are mostly carbonate concretions of permineralized peat that occur in coal seams and contain a rich fossil assemblage of plant debris and invertebrate material. They document the diverse plant assemblages and ecological interactions of the geological past and are a primary source of information about Pennsylvanian (a subperiod of the Carboniferous, 323.2–298.9 Mya) peat swamps (Stopes and Watson, 1909; Phillips, 1980; Scott and Rex, 1985). Coal balls preserve the internal cellular structure of the plants they contain, capturing different stages of the life cycle, including those rarely observed in the compression record. Preserved coal balls often include anatomical and histological structures, including gametophytes (Brack‐Hanes, 1978), apical meristems (Good, 1973; Hetherington et al., 2016), and starch grains (Baxter, 1964), with exceptional specimens displaying the cell contents (Scott and Rex, 1985; Taylor et al., 2009). In addition to their rich paleobotanical composition, coal balls also feature a diverse collection of invertebrate trace material, including borings (Cichan and Taylor, 1982), fecal pellets (Baxendale, 1979; Scott and Taylor, 1983), and other evidence of feeding traces (Scott and Taylor, 1983; Labandeira and Phillips, 1996; Labandeira et al., 1997).

Hooker and Binney (1855) first described coal balls from the Lancashire and Yorkshire coalfields from the Westphalian A (regional Northwest European stage, 313–304 Mya). Many of the early botanical descriptions of coal balls were produced by British researchers and established the foundation for later works. North American coal balls were first described by Noé (1923). Pennsylvanian coal balls are commonly found in both North America and Europe because they were geographically connected as a part of the supercontinent Pangea. Increased funding and interest in natural resources following World War II led to a heightened level of research and “coal ball fever” in North America from 1950 to the late 1980s. During this time, a wealth of data was collected from coal balls originating from the Appalachian region, Illinois, Indiana, Iowa, Kansas, and Texas (Darrah, 1939; Benninghoff, 1942; Andrews, 1951; Andrews and Mamay, 1952; Phillips, 1980). In the 1970s and 1980s, the focus expanded from systematics and comparative morphology to include floristics, community paleoecology, physiology, and diversity (e.g., Phillips et al., 1974; Pigg and Rothwell, 1979; DiMichele and Phillips, 1985; Raymond, 1988), accompanied by the examination of ecological interactions between arthropods and plants (e.g., Scott and Taylor, 1983; Scott et al., 1992; Labandeira and Phillips, 1996).

Coal ball research during the 1950s–1980s introduced several preparation and analysis techniques from different laboratories. However, due to the decline in coal ball research in recent decades, many of these methodologies are not in active use nor discussed in recent publications. In this review, we aim to synthesize previously published techniques and provide a comprehensive contemporary guide to the cellulose acetate peel method and coal ball curation. We also introduce a new protocol for the digitization and curation of peels, which is in use at the Phillips Coal Ball Collection at the University of Illinois Urbana‐Champaign. In documenting these protocols, we hope to establish a standardized procedure for working with coal balls.

## Coal ball formation

Coal balls are often found in situ as isolated masses of carbonate in coal seams. They generally are not found as single, isolated concretions but occur in masses that may be distributed horizontally for many meters or vertically, even encompassing the entire thickness of the seam. They are often oblong spheroids that range in size from a few centimeters to over a meter in length. Formation occurs shortly after the burial of organic material, which is entombed in minerals precipitated from marine water or groundwater, a process known as permineralization (Perkins, 1976). Permineralization most commonly occurs by the precipitation of calcium carbonate, which embeds and thereby preserves the three‐dimensional structure of plant organs and cell walls; dolomite is often part of the carbonate matrix. Coal balls may also be formed through the precipitation of silica, although this is rare (Stopes and Watson, 1909; Feliciano, 1924). Because calcium carbonate precipitation occurs under higher pH conditions, coal balls likely formed where large planar (topogenous) peat deposits were the main peat bodies. Domed (ombrogenous) peats tend to be highly acidic, creating an unfavorable chemical environment for coal ball formation. In contrast, planar peats, or areas of peatland complexes (mixed planar and domed), contain areas of more neutral pH that could be buffered by groundwater or marine water.

Coal ball formation is complex. There is no one widely accepted hypothesis regarding either the source and origin of mineral‐rich waters necessary for permineralization or the timing of that permineralization. The wide variety of coal ball types suggests that there are multiple pathways for forming coal balls, with no one unique formation type (Stopes and Watson, 1909; Mamay and Yochelson, 1962; Perkins, 1976; Scott and Rex, 1985; Scott et al., 1996). Despite this evidence for various conditions leading to coal ball formation, coal balls do not occur in later Mesozoic or Cenozoic coal beds. One area of disagreement is the source of calcium carbonate in coal balls. The earliest geochemical analysis conducted concluded that it originated from a marine source (Stocks, 1902). Shortly after this work, Stopes and Watson (1909) demonstrated that coal balls formed in situ and proposed that seawater acted as the initial preservative and source of calcium carbonate for coal balls. Several studies have argued for an indirect groundwater origin of calcium carbonate as many coal swamps were freshwater systems (Feliciano, 1924; Evans and Amos, 1961; Phillips and DiMichele, 1981).

Another early question was whether the plant material in coal balls was transported or autochthonous. Due to the high fragmentation of the contents contained within coal balls, Lomax (1902) proposed that their contents were transported from their original source prior to formation. Further research demonstrated that coal balls universally occur under marine‐sediment coal‐bed roof strata with much of their contents originating from an autochthonous source (Stopes and Watson, 1909; Holmes and Scott, 1981; Scott and Rex, 1985; Demaris, 2000), indicating that the permineralization of calcium carbonate in peat captures plant organs in situ.

The occasional occurrence of marine organisms in coal balls and the root morphology of some plant groups in North American coal balls (e.g., cordaitaleans; Cridland, 1964) have led some authors to conclude that the environment of coal ball formation resembled that of the mangrove coastal marshes in Southwest Florida, where influxes of marine water introduced sufficient calcium carbonate to foster permineralization (Perkins, 1976). Mamay and Yochelson (1962) first reported coal balls bearing marine animal remains from the Carboniferous assemblages of McAlester, Oklahoma; southeastern Kansas; Oskaloosa, Iowa; and Berryville, Illinois. They described three types of coal balls based on their composition: (1) normal, only containing plant material; (2) mixed, containing plant and marine animal remains; and (3) faunal, containing only marine animal remains. They suggested that mixed coal balls were formed when mud rollers or mud slurries (a mixture of silt and clay containing animal remains) were introduced into swamps by marine inundations such as tidal waves or storms. Later studies demonstrated that most of the marine muds, and associated marine invertebrates, are found in coal balls restricted to the upper layers of coal beds and likely originated as burrows into the peat during marine inundation and termination of peat swamps. Marine coal balls are common in North America, but only one European marine coal ball has been reported, from the Westphalian A of Lancashire, England (Holmes and Scott, 1981).

During the Pennsylvanian, Earth experienced a long cool climate phase, composed of regular glacial–interglacial cycles. Peat formed during the glacial phases of these cycles when seawater was pulled off the continental shelves and stored in grounded ice. The Intertropical Convergence Zone (ITCZ) was narrowly confined to equatorial regions, greatly reducing the seasonality of tropical rainfall (Cecil et al., 2003; Peyser and Poulsen, 2008). It also was a time of cool, perhaps even cold, temperatures in the tropics (23°N–23°S) (Tabor et al., 2013; Matthaeus et al., 2021). Paleogeography and climate created the optimal environment of ever‐wet tropical conditions and a continual depositional system for coal ball formation (Nelsen et al., 2016). During the wetter intervals of glacial–interglacial cycles, significant amounts of tropical forest peat were deposited in central and eastern Euramerica, when North America and Europe were part of the supercontinent Pangea (Chaloner and Lacey, 1973; Ziegler et al., 1981; DiMichele, 2014).

Coastal environments were subject to periodic flooding during the ice‐melting/sea‐level‐rise phase of Carboniferous glacial–interglacial cycles. These cycles appear to have been driven by Milankovitch orbital variations (Montañez and Poulsen, 2013; Raymond et al., 2019). Changes in Milankovitch orbital cycles and a shift to a drier paleo‐tropical climate during the Late Pennsylvanian led to a general decrease in coal‐bed extent and thickness, leading to a decline in coal ball occurrences (Raymond et al., 2019).

## Distribution

The earliest known coal balls are from the mid‐Carboniferous, near the Mississippian–Pennsylvanian boundary in the Ostrava‐Karvina Basin of the Czech Republic (Beckary, 1987; Galtier, 1997), which marks the appearance of widespread glaciation in the Southern Hemisphere (Bouroz et al., 1978; Phillips, 1980; Phillips and Peppers, 1984; Phillips et al., 1985; Galtier, 1997; Rygel et al., 2008). Changes in cyclothem sediments, peat geochemistry, and community composition indicate cycles of a wet to dry paleo‐tropical climate during this interval (Gastaldo et al., 1996; DiMichele et al., 2009; Montañez and Poulsen, 2013; DiMichele, 2014). Coal ball abundance diminished by the early Permian (Asselian–Sakmarian), although glacial cycles continued to persist during the early Permian (Phillips, 1980; Chen et al., 2013; Montañez and Poulsen, 2013). The lack of Mesozoic and Cenozoic coal balls in paleo‐tropical coals, however, reveals that glacial climate is only one of many environmental requirements needed for coal ball formation.

Coal balls have primarily been described from the mid‐Carboniferous to Permian (323.2–252.9 Mya). The majority of the European and Donets Basin coal ball assemblages are Westphalian (313–304 Mya) in age, with North American coal balls being more widespread, representing a larger stratigraphical (late Mississippian to Permian [358.8–252.9 Mya]) and geographical distribution (Snigirevskaya, 1972; Phillips, 1980; Galtier, 1997). Chinese coal balls have been reported from the Namurian of the Pennsylvanian (326 and 313 Mya) to the Changhsingian stage (254.2–252.2 Mya) of the upper Permian representing the floral assemblages of the microcontinent Cathaysia (Li et al., 1995; Tian et al., 1996).

**Figure 1:**
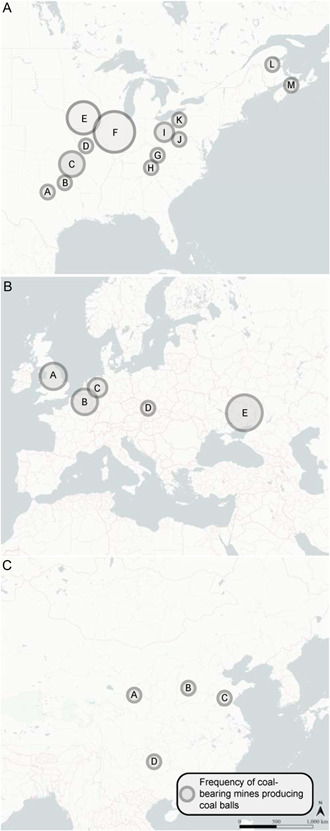
Maps depicting the distribution of coal balls in (A) North America (refer to Appendix 1 for locality names), (B) Europe (refer to Appendix 2 for locality names), and (C) China (refer to Appendix 3 for locality names). Localities are sourced from Phillips et al. (1985) and Tian et al. (1996). The size of the circles indicates the frequency of coal‐bearing mines in a locality; the smallest circle represents fewer than two localities and the largest circle represents more than 15 localities.

Coal balls have been found in over 200 locations including Austria, Belgium, Canada, China, Russia, Ukraine, Germany, the United Kingdom, the Netherlands, Spain, and the United States (Figure 1, Appendices 1., 2., 3.) (Koopmans, 1933; Kindle, 1934; Baxter, 1960; Snigirevskaya, 1972; Phillips, 1980; Scott and Rex, 1985; Galtier, 1997; Lyons et al., 1997). Phillips (1980) provided a comprehensive account of the stratigraphical and geographical distribution of coal balls, which identified five major coal ball regions: (1) the Donets Basin of Russia and Ukraine; (2) Western and Central Europe; (3) the Appalachian region of the United States; (4) the Eastern Interior region of the United States; and (5) the Western Interior region of the United States.

## Petrographic thin sections

One standard method for studying coal balls is the use of thin sections, which has been the standard in paleobotany for over a century. Coal balls were prepared using thin sections until the development and refinement of the cellulose acetate peel method. Petrographic thin sections are thin slices of rock (~30 µm) mounted on a glass slide. These sections are made by cutting rock or mineral samples and mounting each sample to a glass slide with an adhesive. The sample is then ground and polished to a thin flat surface using a series of abrasive grit with decreasing particle size or with specialized machinery. The process of making a thin section is time‐consuming and can take several hours or days. It requires specialized equipment and the process of cutting through the coal balls destroys some material.

Thin sections can be studied using a compound light or polarizing microscope and provide detailed information on wood anatomy or about the mineralogy, texture, and structure of fossil samples. In some instances, such as in the study of fossil wood, they are the primary preparation technique (Taylor et al., 2009). While thin sections have been largely replaced by the acetate peel method in most coal ball research, acetate peels sometimes do not capture enough material for the study of certain plant parts and fungi body structures (Taylor et al., 2011). In such cases, thin sections offer an advantage as they can be cut thicker than acetate peels and capture more three‐dimensional details.

## The peel method

**Figure 2:**
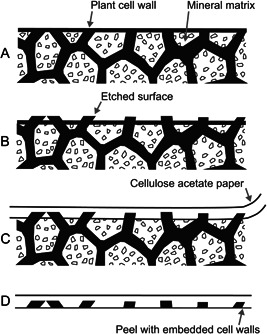
The stages of the cellulose acetate peel method, displaying the transfer of material from a sectioned coal ball to acetate paper. (A) The surface of a sectioned coal ball. (B) The surface of a coal ball has been etched with acid to release organic material slightly above the surface from the mineral matrix. (C) The etched surface of the coal ball is flooded with acetone, and acetate paper is applied to the coal ball's surface where it is softened sufficiently by the acetone to entomb the projecting organic matter. (D) Once dried, the peel is removed to reveal plant cell walls from the coal ball embedded in the cellulose acetate. (Modified with permission from Lacey, 1963, fig. 6.9 and Galtier and Phillips, 1999, fig. 13.1).

Cellulose acetate peels are a faster alternative to petrographic thin sections. An acetate peel captures a ~30‐µm organic layer from the cut surface of a coal ball (Figure 2). The peel captures individual cells and can be used to identify plants and produce a taxonomic census of coal ball assemblages, thus serving as a critical data source for paleoecological analyses of coal balls. Additionally, peels can be used to study whole‐plant organs, offering insights into gross plant anatomy and cellular structure, from which aspects of physiology can be inferred (see Wilson and Knoll, 2010; Wilson et al., 2015). While thin sections can also document these structures, peels capture the entire face of a sectioned coal ball, are permanent, and are easy to store.

The peel method has been refined over time. The technique was first described by Walton (1928) in collaboration with R. G. Koopmans. The surface of a sectioned petrification was polished and etched with a dilute acid (commonly hydrochloric acid [HCl]). A solution of cellulose ester was poured onto the etched surface, which dried to a film that captured material from the surface and could be peeled. Graham (1933) adapted this technique for coal balls from the Illinois Basin, using a solution of nitrocellulose, which reduced the number of air bubbles and adhesion to the surface of the coal ball. Because these techniques used a liquid solution, they became known as the liquid‐peel method.

Joy et al. (1956) replaced the liquid peel with cellulose acetate paper, significantly reducing the drying time associated with cellulose esters. Mounted acetate peels showed less contraction (<1%) compared to peels produced using the method of Walton (1928) (2.0–2.5% vs. 4.0–4.5% contraction over 8 years) (Joy et al., 1956). Contraction in acetate peels typically takes place within the first 48 h after peeling and continues slowly over time (Joy et al., 1956).

## PROTOCOL FOR CELLULOSE ACETATE PEELS AND METHODOLOGICAL CONSIDERATIONS

**Figure 3:**
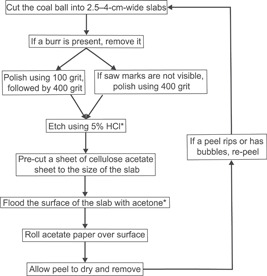
A flowchart summarizing the cellulose acetate peel method. *Designates the use of a fume hood or personal protective equipment.

The peel protocol that follows is modified from Joy et al. (1956), Lacey (1963), Phillips et al. (1976), and Galtier and Phillips (1999). We synthesize and update these techniques and include specific considerations in preparing peels (Figure 3).

### Mechanical and chemical safety

The mechanical and chemical processing needed for coal ball preparation and curation requires supervised training. The rock saws used to section coal balls are powerful tools that should be used with caution. Always wear eye and hearing protection when operating a saw. Ensure that the sample is firmly secured in the vise prior to cutting and use a slow steady feed rate to avoid forcing the blade through the material. Do not handle the saw blade when it is on or leave the saw unattended.

The chemicals used in our protocols pose serious health hazards. Proper lab safety should always be followed when preparing coal balls. Users should familiarize themselves with the material safety data sheet for each chemical before beginning. Protocols and safety equipment should be reviewed and approved by your institution's department of research safety. Chemicals should always be used in a fume hood while wearing the appropriate personal protective equipment, including a lab coat, apron, protective gloves, and goggles.

HCl is a strong and corrosive acid that should be added to water slowly and not the other way around. Always wear gloves when working with HCl even if dilute. Xylene is a colorless flammable liquid that is potentially lethal with high exposure. Hydrofluoric acid (HF) is a colorless solution of hydrogen fluoride in water. It is an extremely hazardous acid that will dissolve glass and other silicates and can be lethal if it is inhaled, ingested, or comes into contact with the skin. The use of HF requires specialized training and facilities for safe handling. Personal protective equipment for working with HF includes a face shield, neoprene and latex gloves, a rubber apron, coveralls, chemically resistant booties, and eye protection. Use only polyethylene containers and carefully place specimens to avoid splashing. Emergency equipment including an eye wash, shower, chemical spill kit, and calcium gluconate gel should be on hand before beginning.

### Cutting a coal ball

Coal balls can be sliced into 2.5–4‐cm‐thick slabs using an oil‐cooled lapidary saw in a fashion similar to slicing sandwich bread. Water‐cooled lapidary saws are not recommended as they accelerate the deterioration of the coal ball by forcing water into pore space, accelerating pyrite oxidation. The use of petroleum‐based lubricating oils precludes coal balls from a range of geochemical and organic chemical analyses. Chipping or fracturing the coal ball with a hammer and chisel is recommended if geochemical sampling is the primary focus.

Because coal balls are oblong spheroids, the first cut should be made along the longitudinal median axis (Appendix S1). After every cut, examine each slab and readjust the orientation or angle of the coal ball to cut along the centerline of any plant organ of interest. Once cutting is completed, thoroughly wash each slab with warm water, detergent, and a brush to remove all traces of cutting oil. This will prevent oil from penetrating the slab, which could cause the coal ball to become brittle. Residual oil also will reduce the quality of peels that can be made from the coal ball, even making it impossible to produce a peel.

It is important to keep in mind that not all coal balls are uniformly shaped. This is especially true if the coal balls were in situ and removed directly from the coal face, as during the collection of vertical profiles. In these cases, the use of picks, chisels, and rock hammers can fracture coal balls, resulting in uneven shapes with sharp edges and fractures.

### Trimming the slab

After a coal ball is cut using a lapidary saw, a small burr may be left at the end of the cut. This burr will interfere with polishing and the peeling process. To remove the burr, carefully chip or grind it off using a hammer and chisel, nipping pliers, bench grinder, or bastard file. Remove all protrusions that would prevent the slab from having a flat surface.

### Polishing the surface of a sectioned coal ball

A smooth, level surface prevents pooling of acetone, which can cause wrinkling of the cellulose acetate paper when the paper is applied to the etched surface. To polish the surface of a sectioned coal ball, use a lap wheel or glass plate (>6 mm thick) with a slurry of carborundum powder (silicon carbide) and water. To make the slurry, wet the surface of the glass plate or a lapidary wheel with water using a standard laboratory gooseneck plastic spray bottle. Sprinkle powdered carborundum grit onto that surface to create a soft, but not flowing, liquid mixture of water and carborundum. Grit can be kept in shakers to simplify its application.

Begin with 100 grit to remove all traces of saw marks. For 0.005‐inch (0.127 mm) cellulose acetate paper, transition to 400 grit to polish the surface until it is smooth to the touch. For 0.003‐inch (0.076 mm) cellulose acetate paper, continue polishing with 600 and 800 grit. Wash the surface of the grinding wheel or glass plate between changes in grit size. Ideally, each grit size should have its own glass plate.

Polishing should be performed using a circular or figure‐eight motion, adding pressure to the slab as dictated by the situation. For example, when grinding an undulated or uneven surface on a grinding lap in order to flatten the surface of a coal ball slice, increased pressure will decrease the time necessary for the task. Even in this case, however, the coal ball should be moved continuously over the surface of the wheel. For polishing with the very finest grit particle, gentle pressure may be all that is necessary. Keep in mind that the carborundum is grinding not only the surface of the coal ball, which is relatively soft, but also the surface of the glass plate, which will be made of harder material but still is subject to erosion. Once completed, thoroughly wash the coal ball to remove all traces of grit.

### Etching the surface and drying

Wearing nitrile gloves and using a fume hood, immerse the surface of the slab to be peeled in 5% HCl for 10–65 s (refer to the section on Mechanical and Chemical Safety), using only enough acid to cover the surface to be peeled. The etching time will vary depending on the amount of calcium carbonate and other minerals, such as dolomite, in the coal ball, which will differ by locality. Some experimentation is needed to determine the best etching time for a specific coal ball deposit. A coal ball composed of dolomitic cement will require several minutes of etching or will not etch at all. The more dolomite that is present in a coal ball, the longer the etching time.

Do not touch the etched surface. HCl dissolves calcium carbonate and exposes ~30 μm of organic material. This material is very delicate and can be wiped off with the slightest touch. If this occurs, the etching process must be repeated. When not in use, the container of HCl should be tightly sealed as fumes will rust items within the vicinity. Once etching has been completed, wash the coal ball under running water without touching the etched surface and allow it to air dry on a gravel pit with the etched side face up until completely dried. This process can be accelerated by using compressed air, or even a hairdryer, to dry the sectioned surface. Another option is to flow ethyl alcohol or acetone over the surface several times to accelerate evaporation. However, the best option is compressed air. If the slab is not completely dried, the exposed fossil plant material and cellulose acetate paper will not properly adhere. This will lead to an insufficient amount of material being transferred to the acetate paper and a milky or opaque stain forming wherever water was present.

A gravel pit can be used to securely hold slabs during air drying. The loose gravel accommodates uneven surfaces and allows a slab to sit flat. The pit should be ~4‐cm deep with clean, smooth pebbles ~6 mm in diameter. When peeling both sides of a slab, keep as much of the peeled surfaces off the gravel by resting it on the uncut side surface.

### Siliceous permineralization

Siliceous permineralization requires the use of HF (25–40%) to dissolve the silica (refer to the section on Mechanical and Chemical Safety). The use of HF to make peels follows the application of HCl. After etching has been completed, immerse the specimen in a sodium carbonate solution to neutralize the acid before rinsing with water. The acidity of the surface/sides of the coal ball should be checked with litmus paper to ensure that all HF has been neutralized. Due to the risk associated with the use of HF, a safer alternative is to prepare siliceous permineralization using petrographic thin sections. If a laboratory is not equipped for the use of HF, then thin sections are the only option for processing siliceous permineralizations.

### Applying and removing the cellulose acetate paper

Pre‐cut a sheet of acetate paper to the size of the slab, allowing for ~2 cm of overlap from the edge. With the specimen leveled in a well‐ventilated area or fume hood, flood the surface of the slab with acetone from a gooseneck wash bottle.

Slightly bend the acetate paper in half (do not fold) and delicately roll it over the surface, avoiding the formation of air bubbles. As the paper is rolled over the surface, it will push excess acetone outwards. This must be done rapidly as acetone evaporates quickly. If acetone evaporates before the process is complete, quickly add more acetone to the dry areas and continue rolling the paper over the surface. It is important to roll the acetate paper onto the coal ball; do not drop or press down as this will lead to air bubbles. Once the paper touches the flooded surface, do not touch or readjust the acetate paper.

Too much acetone can break the acetate's crystalline structure, resulting in wrinkling. The proper amount of acetone should be used to enable plant tissue to be embedded onto the acetate paper. This amount can be determined through trial and error. Air bubbles will prevent the transfer of plant material onto the acetate paper and prevent the identification of material; therefore, peels with a substantial number of bubbles must be redone.

**Figure 4:**
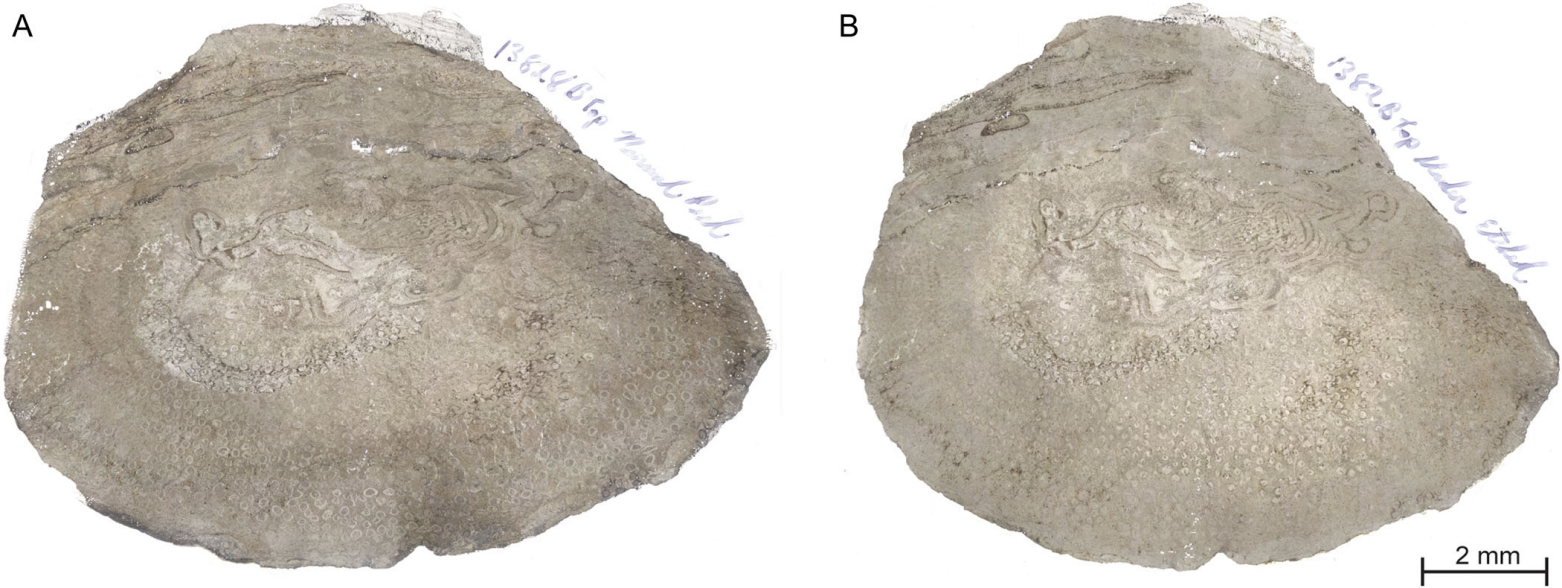
A comparison of peels taken from the same coal ball (University of Illinois Urbana‐Champaign [UIUC] 13828B Top) from the Calhoun Coal, made at different qualities. This peel captures a cross‐section of a *Psaronius* stem (center of peel) and inner root mantle (lower region of peel). (A) A peel that has been etched for the optimal amount of time depicts a greater definition between each root in the inner mantle and stem. (B) A peel that has been under‐etched depicts less definition between each root in the inner mantle and stem.

Wait at least 8 h (preferably overnight) for the acetone to evaporate before removing the peel. Remove the peel by slowly lifting it at one end and gently pulling it away from the coal ball. If desired, the excess acetate paper can be trimmed. Peels that are removed prematurely (before they are sufficiently dry) tend to curl or do not capture enough material. If a peel sticks to the surface, use a razor blade to aid in its removal. A peel that is difficult to remove or tears when peeling has been etched for too long. Peels should capture enough material from the coal ball's surface to clearly identify plant structures (Figure 4A). If the material in a peel appears translucent or it is difficult to discern structures, then the coal ball was under‐etched, or the peel was not allowed enough time to cure (Figure 4B).

### Serial peels and sectioned surface preservation

Peels can be taken consecutively from the same surface, in a process known as serial peeling. The preparation required to re‐peel a surface is dependent on the project goals or how delicate the structure of interest is. If examining a very small structure in fine detail, no surface preparation between peels is needed other than washing the peeling surface with a soft brush or sponge. If examining larger structures, the specimen should be repolished on a grinding plate with 600 grit, using one or multiple figure‐eight motions. As many as 700–800 peels can be made from a 1‐inch‐thick coal ball slab, depending on the etching time. When performing serial peels, the portions of the coal ball that are not being peeled should be capped with latex paint or liquid rubber to protect them from excess acid.

### Types of cellulose acetate paper

Cellulose acetate paper comes in a variety of thicknesses and is described using standard measurements. The two thicknesses primarily used in making peels are 0.005 inches (0.127 mm) and 0.003 inches (0.076 mm). Acetate paper with a thickness of 0.005 inches is used to make the first peel of a coal ball after it has been cut with a rock saw. This paper type can also be used for test peels to identify the optimum etching time in acid. The 0.005‐inch‐thick acetate paper is more robust, reducing the chance of damage when removed. Acetate paper with a thickness of 0.003 inches is used to make peels for microscopic examination, slide mounting, or digitization. The thinner paper allows more transmittance of light, increasing optical resolution. Due to its thinness, this paper is more susceptible to bubbles and wrinkling. A razor blade may be required to remove the peel from the face of a coal ball.

## PROTOCOL FOR SLIDE MOUNTING A PEEL

**Figure 5:**
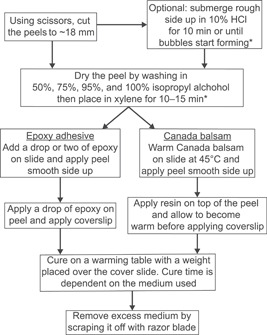
A flowchart summarizing mounting a peel onto a glass slide. *Designates the use of a fume hood or personal protective equipment.

Portions of a peel can be sectioned and mounted onto standard microscope slides to observe finer‐scale features, such as microbes or fungi, at higher magnification (Figure 5) (Taylor et al., 2011). Mounted peels may also be useful for identifying arthropod fecal pellets and their macerated contents. Excess calcite in strongly permineralized material is removed by the mounting process, improving visual resolution.

### Cutting the peel

Using scissors, cut out the area of the peel to be mounted. The section should be slightly smaller than the coverslip (~18 × ~18 mm, but no less than ~15 × ~15 mm). A standard glass microscope slide (22 × 75 mm) can be used for mounting. Larger nonstandard slides and coverslips are available for the mounting of larger specimens.

### Clearing the trimmed peel

Excess calcite, moisture, and debris can be removed to increase the optical resolution of the peel. The following steps must be completed in a fume hood with the appropriate personal protective equipment (refer to the section on Mechanical and Chemical Safety). Using tweezers to handle the trimmed peel, submerge the peel section rough side up in 10% HCl for up to 10 min or until bubbles stop forming. Rinse the trimmed peel in a container of water to remove the acid and dehydrate it by rinsing with a series of washes of 50%, 75%, 95%, and 100% isopropyl alcohol. Do not blot dry the trimmed peel using a paper towel, as this has the potential to introduce paper fibers or dust to the sample.

After drying, submerge the trimmed peel in xylene for 10–15 s to remove all traces of water, then remove the peel from the xylene and allow the excess liquid to drip off. If left in xylene for more than a minute, the trimmed peel will overly harden and become difficult to mount. Peels should be mounted immediately following the use of xylene.

### Mounting the peel using an epoxy adhesive

We recommend mounting the peels using an epoxy adhesive. Place a couple of drops of Opti‐tec 5001 (Intertronics, Kidlington, United Kingdom), Euparal Microscope Slide Mounting Medium (Hempstead Halide, Cambridge, United Kingdom), or a non‐refractive epoxy adhesive of your choice on the center of a glass microscope slide. Place the peel in the center of the slide with the smooth side up. Lower it gently onto the drop of epoxy adhesive, allowing the mounting medium to spread. Place a few drops of epoxy adhesive on the top of the mounted peel. Using forceps, place the edge of the coverslip on the glass slide and slowly lower it onto the mounted peel, allowing the epoxy adhesive to spread. The amount of adhesive needed will vary depending on the size of the peel being mounted. For a standard 22‐mm coverslip, one drop of epoxy adhesive is sufficient. More epoxy adhesive is required if mounting a trimmed peel using larger coverslips.

### Mounting the peel using Canada balsam

An alternative mounting medium is Canada balsam, the resin of the balsam fir that can come in a solid or semi‐solid form dissolved in xylene (refer to the section on Mechanical and Chemical Safety in the Protocol for Cellulose Acetate Peels). The solid form, which is less readily available, will need to be melted on the slide before applying the peel. We recommend working with the semi‐solid form.

Protect the surface of a hot plate by covering the top surface with aluminum foil. Set the hot plate to 45°C, and place a glass microscope slide with a small amount of Canada balsam in the center. Once the Canada balsam has warmed, place the peel in the center of the slide with the smooth side up. Lower it gently onto the Canada balsam, allowing the mounting medium to spread. Place a small amount of Canada balsam on top of the mounted peel. Once melted, place the edge of the coverslip on the glass slide and slowly lower it onto the mounted peel, allowing the adhesive to spread. The amount of Canada balsam needed will vary depending on the size of the peel being mounted and the coverslip. More epoxy adhesive is required if mounting a trimmed peel with a larger coverslip. If the Canada balsam spreads onto the sides and bottom of slides, scrape off with a razor blade after it has dried.

While Canada balsam has historically been the mounting medium of choice for peels, slides using this medium tend to chemically age, with increasing coloration or yellowing that interferes with observation and imaging (Griffith, 1843; Bracegirdle, 1989; Schmid et al., 2021). Modern mounting media such as Opti‐tec 5001 and synthetic Canada balsam may be more stable for long‐term storage, but studies are lacking. Other mounting media (e.g., Permount, Fisher Scientific, Waltham, Massachusetts, USA) show signs of physical deterioration (cracks) within a few years (Schmid et al., 2021). The purpose of the peel and its archival life should dictate the mounting medium used.

### Curing

Depending on the mounting medium, cure times will vary. Check the product instructions for further instructions on curing. Some adhesives may require a warming table or oven to accelerate the curing time. If using Opti‐tec 5001, curing can be done at room temperature for 24 h or at 65°C for 1 h. Some mounting media such as Canada balsam (solid or semi‐solid) must be cured by placing the slide on a warming table or hot plate at 38°C for several days or weeks. To ensure that the mount is flat and free of air bubbles, place a metal weight with a base smaller than the coverslip while it cures. Lead weights used for scale calibrations are ideal.

### Repairing degrading coal balls

Coal balls typically contain cracks, which can cause problems with long‐term storage or handling. These cracks are weak points along which fractures can form if enough pressure is applied. Fractured coal balls and slabs can be repaired by using water putty to join the pieces back together. The exposure of coal balls to atmospheric O_2_ and moisture promotes the oxidation of pyrite (FeS_2_) contained within coal balls, forming sulfur dioxide (SO_2_), and accelerating their degradation. The process can be slowed by embedding the sample in liquid plastic or resin. Once a coal ball shows signs of degradation due to pyrite oxidation, the progression can only be slowed, not stopped.

### Repairing fractures with water putty

Mix water putty powder with water in a disposable container according to the instructions. Place the fractured coal ball slab on a flat surface and moisten the fractured faces with water. Dab the water putty paste onto the fractured faces using wooden popsicle sticks and press the faces firmly together. Clean off excess paste with a damp paper towel. This process can also be applied to whole coal balls that have fractured before they are cut into slices.

### Embedding coal balls in liquid plastic or resin

Plastic or resin can be used to repair a coal ball or slab that has been highly fractured or to protect it from pyrite oxidation. Reassemble the fragmented coal ball or slab and glue the pieces onto a glass plate (>6 mm thick) that extends several inches from the mounted specimen on all sides. Allow for at least 6 mm of space between fragments for the embedding medium to strengthen the mount, as plastic or resin will shrink as it dries. If too little embedding medium is present between fragments, the mount can split.

Make a mold for the embedding medium by using a cardboard box that allows for 1.2 cm of space between the sides and top of the specimen mounted onto the glass plate. Line the box with aluminum foil and pour the mixed liquid into the mold with the mounted specimen. Use as little embedding medium as possible, as acetate peels will not stick to the medium. Submerge the specimen in the embedding medium with at least 6 mm to 2.5 cm of the coal ball's surface protruding. Specimens that are highly fractured or degraded will require more of the specimen to be embedded in epoxy.

Once the slab or coal ball is embedded, allow it to cure undisturbed for 48 h or until the medium has hardened. Remove the embedded mount from the mold. If desired, the glass plate can be removed by twisting the embedded coal ball or slab from the plate. Trim off all sharp plastic or resin edges using cutting pliers or sandpaper.

### Encasing coal ball slabs in paraffin

Paraffin can also be used to slow the degradation of coal balls and sectioned slabs. The method was developed and used at the Cleveland Museum of Natural History (Chitaley, 1985). A slab that has been polished on both sides should be baked in an oven at 250°C to remove all traces of moisture. Submerge the slab in ethyl alcohol for 5 min to kill all sulfate bacteria. Once cooled, immerse the slab in xylene for 2 min and then through a serial solution of 2%, 5%, and 15% solutions of paraffin in xylene (refer to the section on Mechanical and Chemical Safety). Allow the slab to sit in each solution for several minutes to enable penetration of the paraffin. Embed the slab completely in melted wax using a mold constructed slightly larger than the specimen. Depending on the size of the slab, disposable plastic containers make suitable molds. Using a sharp blade, remove the surface layer of wax to expose the surface and rinse with xylene. The surface should then be polished using 1000 grit with ethyl alcohol as the liquid carrier for the grit solution and rinsing. It is vital to not expose the wax‐encased slab to water as this will lead to the degradation of the sample.

Treat the surface with 70% nitric acid for 5–15 min, depending on the material type. Rinse the specimen with ethyl alcohol until the acid is completely washed off, checking with a piece of litmus paper. Using 0.005‐inch cellulose acetate paper, make a peel of the slab's surface and observe under a microscope. If the specimen's surface provides enough detail, cover it with a new piece of acetate for storage. If the peel is under‐etched, polish the surface lightly with 1000 grit and etch again before trying another peel. Re‐infiltrate the surface with wax every 5–10 peels, depending on the condition of the slab.

## CURATING COAL BALLS AND PEELS

### Storing coal balls

The high percentage of pyrite in coal balls can cause them to disintegrate over time if they are not properly stored. To prevent pyrite oxidization, keep coal balls in a well‐ventilated area in a container that allows for airflow. Canvas bags and wooden boxes with loosely fitted covers are ideal for storage; avoid storing coal balls in sealed containers where airflow is limited. Our experience with the University of Illinois Phillips Coal Ball Collection suggests that temperature and humidity fluctuations may be beneficial, as they enable coal balls to outgas. Subjecting a coal ball to constant temperature and humidity will promote degradation. To further protect the sectioned surface, coal balls can be stored with 0.005 inches of cellulose acetate paper mounted to both sides of each slab.

### Storing peels

For storage, peels should be taped or paper‐clipped smooth side up to an index card or sheet of stiff white paper. Prior to mounting a peel to a piece of paper, the peel should be labeled with an identifying specimen number or other unique identifiers and the excess acetate paper should be trimmed with scissors. Peels mounted this way can be stored in a binder or envelope. Unmounted peels may contract and fold, and improperly stored peels will tend to curl over time. Peels can be flattened by using a hot‐roller laminator set at 100°C (Institute of Geosciences, Division of Paleontology, University of Bonn, Germany); they should be placed in the laminator protective sleeve when being processed by the laminator. For long‐term archiving, acid‐free paper and archival pen ink should be used.

## DIGITIZATION OF PEELS

### Scanning with a flatbed scanner

Peels can be quickly and efficiently digitized using a flatbed scanner. We currently use both an Epson Perfection v850 Pro (Epson, Tokyo, Japan) and Canon CanoScan 9000 F (Canon, Tokyo, Japan). However, almost any high‐quality flatbed scanner with a charge‐coupled device (CCD) array element capable of a hardware‐native 800–1200 dpi imaging resolution will be sufficient. When assessing potential scanners, note that advertised scanning resolutions in excess of 1200 dpi are often produced using digital interpolation and not higher‐resolution scanning. These extra pixels are produced algorithmically, usually an average of the adjacent pixels, creating the perception of a higher‐resolution image. Best practice is not to exceed the hardware resolution of the scanner.

Place the peel smooth side down onto the flatbed scanner with a white sheet of paper as the backdrop. We recommend adding identifying information, metadata, etc., in the white space collar area of the backing paper. While adding this content increases the overall size of the resulting digital image file and increases preparation time, it also permanently attaches important archival information to the image itself. The peel should then be scanned at a minimum resolution of 800 dpi or greater. Increasing the resolution will increase the amount of time needed per scan, although the differences may be nominal if hardware CCD limits are not exceeded and the host computer, scanner, and connecting cables are of recent vintage. Overall, in our experience, with our scanning hardware, we found scanning at 1200 dpi (the maximum hardware resolution of our scanner CCD elements) to be worth the increased scanning time.

### Scanning with a camera‐mounted dissecting microscope

**Figure 6:**
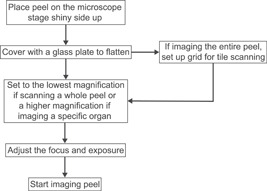
A flowchart summarizing scanning a peel with a camera‐mounted dissecting microscope.

Motorized stereo‐zoom microscopes produce archival‐quality images of peels at high magnification. We use a Zeiss Axio Zoom V16 microscope and Zeiss Zen microscopy software (Zeiss, Oberkochen, Germany). Transmitted light sources work best for illumination, as peels are translucent. External light sources such as an illumination ring or side lights are not recommended as they could cause glare. An entire peel can be digitized using tiling software, which captures multiple images of the peel in a gridded pattern (image tiles) and stitches them together to create one composite image of the entire peel (Figure 6). Image tiles should overlap by 10% for auto‐stitching. Place a glass plate over the peel to keep the peel flat while imaging. Use the lowest magnification (7×).

### Flatbed scanner vs. high‐magnification microscopy

**Figure 7:**
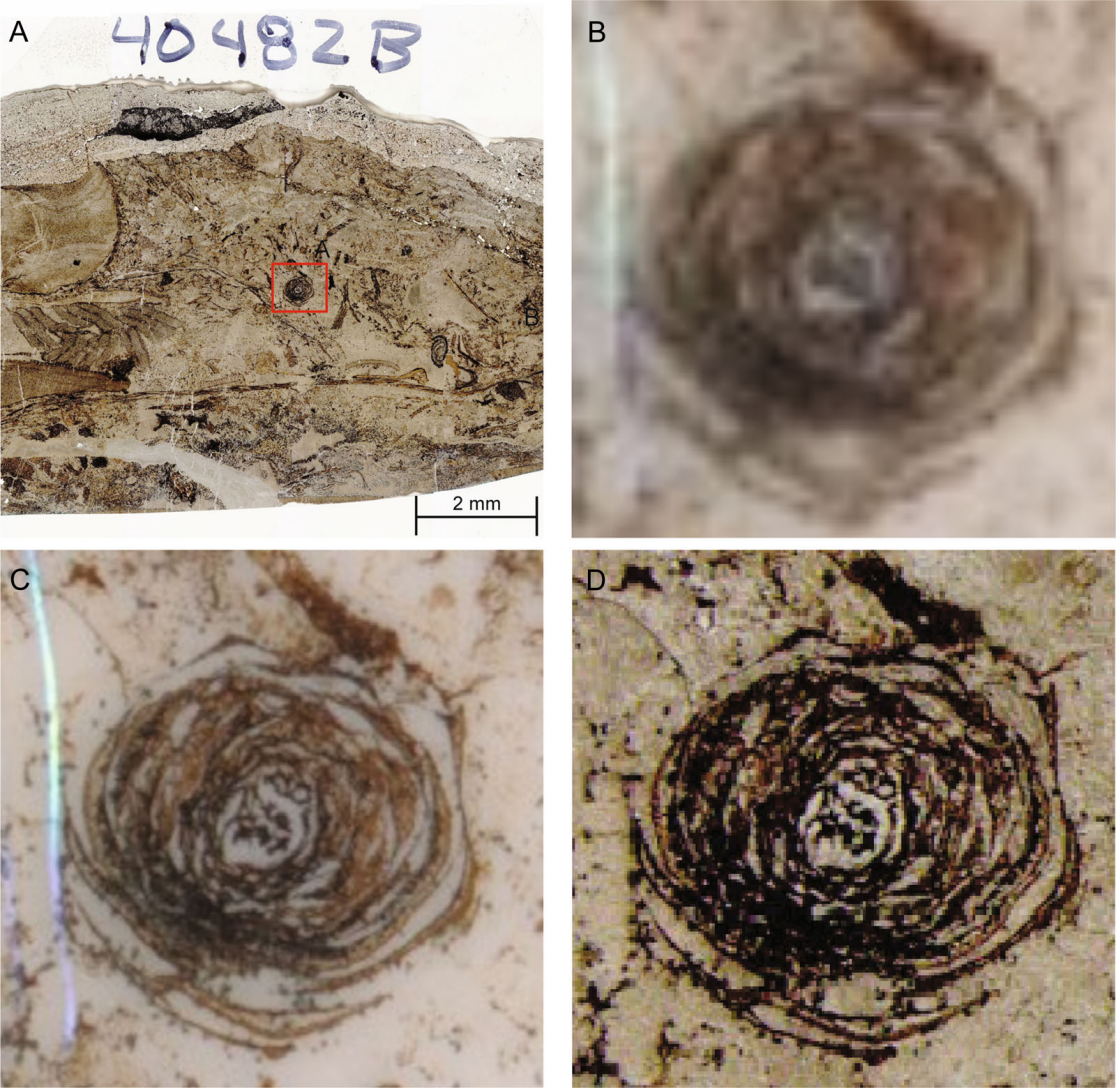
A comparison of digitized peels using flatbed scanning and high‐magnification microscopy. (A) Peel UIUC 40483B from the Mt. Rorah Coal with a cordaitean strobilus circled in red digitized at 2500 dpi using an Axio Zoom V16. (B) A flatbed scan at 800 dpi of peel UIUC 40483B focused on the cordaitean strobilus. (C) A flatbed scan at 1200 dpi of peel UIUC 40483B focused on the cordaitean strobilus. (D) A high‐magnification scan taken with an Axio Zoom V16 at 2500 dpi of peel UIUC 40483B focused on the cordaitean strobilus.

Both flatbed scanners and stereomicroscopes render a digital version of a peel, but the resolution will be very different. Flatbed scanners are most useful for the quick digitization of a whole peel, where more detail and smaller structures are not the main focus of the imaging project (Figures 7B and 7C). Digitized peels that are intended for publication, teaching, or archival purposes should be imaged using high‐magnification microscopy, as the higher resolution is needed to capture the morphology of finer structures (Figure 7D). In most applications, we recommend using high‐magnification microscopy for digitization if available.

### File types and data storage

Selecting the right file type is important for preserving image resolution and metadata. For flatbed scanners, PNG files are optimal as they use lossless compression and remove significant amounts of data redundancy within an image. They tend to have smaller file sizes compared to other file types and are an open format that can be read by many types of software. Files produced by high‐magnification microscopy tend to be large, ranging from 300 MB to 20 GB depending on the size of the peel being scanned. Larger peels will produce a larger file type and vice versa (~22 cm^2^ = 1 GB).

TIFF is considered an image archival standard. It is an Adobe open format and American National Standards Institute (ANSI) standard. However, file sizes can be large (>30 GB). Another option is to save using the original microscopy format. An advantage of these files is that the metadata and image are saved in a highly compact form. However, microscope image file formats are typically proprietary to the microscope and its software, and researchers should consider whether open‐source software such as ImageJ or Python packages for open microscopy can open these proprietary formats. An alternative is to export images to an open format, e.g., Digital Imaging and Communications in Medicine (DICOM).

Images produced with the support of federal funding should be published in public data repositories. Many museums and universities have their own public‐facing image databases, such as the Illinois Data Bank at the University of Illinois (https://databank.illinois.edu/) and the Smithsonian Digital Asset Management System (https://www.si.edu/dams). Perhaps the best general resource for the biological imaging community is MorphoSource (https://www.morphosource.org/), a public image platform for a large range of biological specimens (Boyer et al., 2017). While MorphoSource originated as a repository for computed tomography and magnetic resonance imaging scans of vertebrate skeletal material, it now hosts a range of biological images in a variety of open image formats.

When archiving images, researchers should follow Darwin Core metadata standards for specimen identification (Wieczorek et al., 2012) and Audubon Core metadata standards for biological specimen media (Morris et al., 2013). Following these standards will ensure that critical information needed for future interpretation and analysis of archived images is preserved.

## CONCLUSIONS

Coal balls remain a large source of untapped paleobotanical data. With fewer coal ball researchers active in the field today than in previous decades, documenting current best practices is critical to training a new generation of experts. Despite a long and productive research history, many open questions remain on the formation, botanical content, morphological classifications, and ecological interpretations of coal balls. New digitization technologies hold the potential to facilitate new research areas and public outreach opportunities. Digitization expands access while preventing the risk of further degradation or loss during transport. Digitized collections increase the potential pool of researchers with access to paleobotanical material, including expanding opportunities for students to gain experience with a wide range of specimen types. Morphology‐centered research, like the analysis of coal ball peels, will benefit from these emerging digitization approaches.

## AUTHOR CONTRIBUTIONS

S.R.L. conceived the research and wrote the manuscript; S.W.P. contributed to the writing of the manuscript and development of microscope imaging protocols and supervised the research; S.E. contributed to the flatbed scanning digitization protocol. All authors approved the final version of the manuscript.

## Supporting information


**Appendix S1.** An illustration of how to slice a coal ball. Slabs (2.5–4 cm thick; represented by dashed black lines) should be cut perpendicular to the coal ball's longitudinal median axis (solid gray line).

## Data Availability

All supporting data and information are provided with the article.

## Appendix 1.

### A list of the coal ball–bearing localities in North America identified in Figure 1A.

**Table:**

Locality on map	Coal bed name	Location
A	Newcastle Coal	Texas, USA
B	Secor Caol	Oklahoma, USA
C	Coal below Hogshooter Ls.	Oklahoma, USA
C	Iron Post Coal	Oklahoma, USA
C	Mineral & Iron Post Coals	Oklahoma, USA
C	Mineral & Fleming Coals	Kansas, USA
C	Weir‐Pittsburg Coal	Kansas, USA
C	Bevier Coal	Kansas, USA
C	Blue Jacket Coal	Missouri, USA
D	Above Tebo Coal	Missouri, USA
E	Rock Island Equivalent	Iowa, USA
E	Unnamed Cherokee Group Coal	Iowa, USA
E	Buffaloville Equivalent	Iowa, USA
E	Rock Island & Buffaloville Equivalents	Iowa, USA
F	Lower Block Coal	Indiana, USA
F	Buffaloville Coal	Indiana, USA
F	Murphysboro Equivalent	Indiana, USA
F	Parker Coal	Indiana, USA
F	Colchester (No. 2) Coal	Illinois, USA
F	Summum (No. 4) Coal	Illinois, USA
F	Springfield (No. 5) Coal	Illinois, USA
F	Briar Hill (No. 5A) Coal	Illinois, USA
F	Herrin (No. 6) Coal	Illinois, USA
F	Danville (No. 7) Coal	Illinois, USA
F	Unnamed Coal	Illinois, USA
F	Friendsville Coal	Illinois, USA
F	Opdyke Coal	Illinois, USA
F	Calhoun	Illinois, USA
F	Unnamed coal member of the Shumway Cyclothem	Illinois, USA
F	DeKoven Coal	Kentucky, USA
F	Baker Coal	Kentucky, USA
G	Copland Coal	Kentucky, USA
H	Splint Coal	Tennessee, USA
I	Redstone or Pittsburgh Coal	Ohio, USA
I	Meigs Creek Coal	Ohio, USA
I	Ames & Duquesne Coals	Ohio, USA
I	Loer Freeport Coal	Ohio, USA
J	Pittsburgh Coal	West Virginia, USA
K	Coal above Middle Kittanning	Pennsylvania, USA
L	Pictou Coal	New Brunswick, Canada
M	Stellarton Formation	Nova Scotia, Canada

## Appendix 2.

### A list of the coal ball–bearing localities in Europe identified in Figure 1B.

**Table:**

Locality on map	Closest town's name	Country
A	Lancashire & Yorkshire	United Kingdom
B	Bray	Belgium
B	Wėrister	Belgium
B	Juplille	Belgium
C	Kerkrade & Aachen	Netherlands
D	Würm	Germany
D	Duisberg	Germany
D	Langendereer	Germany
D	Orlova & Ostrava	Czech Republic
E	Donets Basin	Ukraine

## Appendix 3.

### A list of the coal ball–bearing localities in China identified in Figure 1C.

**Table:**

Locality on map	Closest city's name	Province
A	Jingyuan	Gansu
B	Taiyuan	Shanxi
C	Zhaozhuang	Shandong
D	Shuicheng County	Guizhou
