# Supplementary material for: Review of the cellulose acetate peel method and the physical and digital curation of coal balls
Source: Appl Plant Sci. 2023 Nov 29;11(6):e11556. doi: 10.1002/aps3.11556 (PMC10719873; doi:10.1002/aps3.11556)

DOI 10.1002/aps3.11556

**APPENDIX S1.** An illustration of how to slice a coal ball. Slabs (2.5–4 cm thick; represented by dashed black lines) should be cut perpendicular to the coal ball's longitudinal median axis (solid gray line).

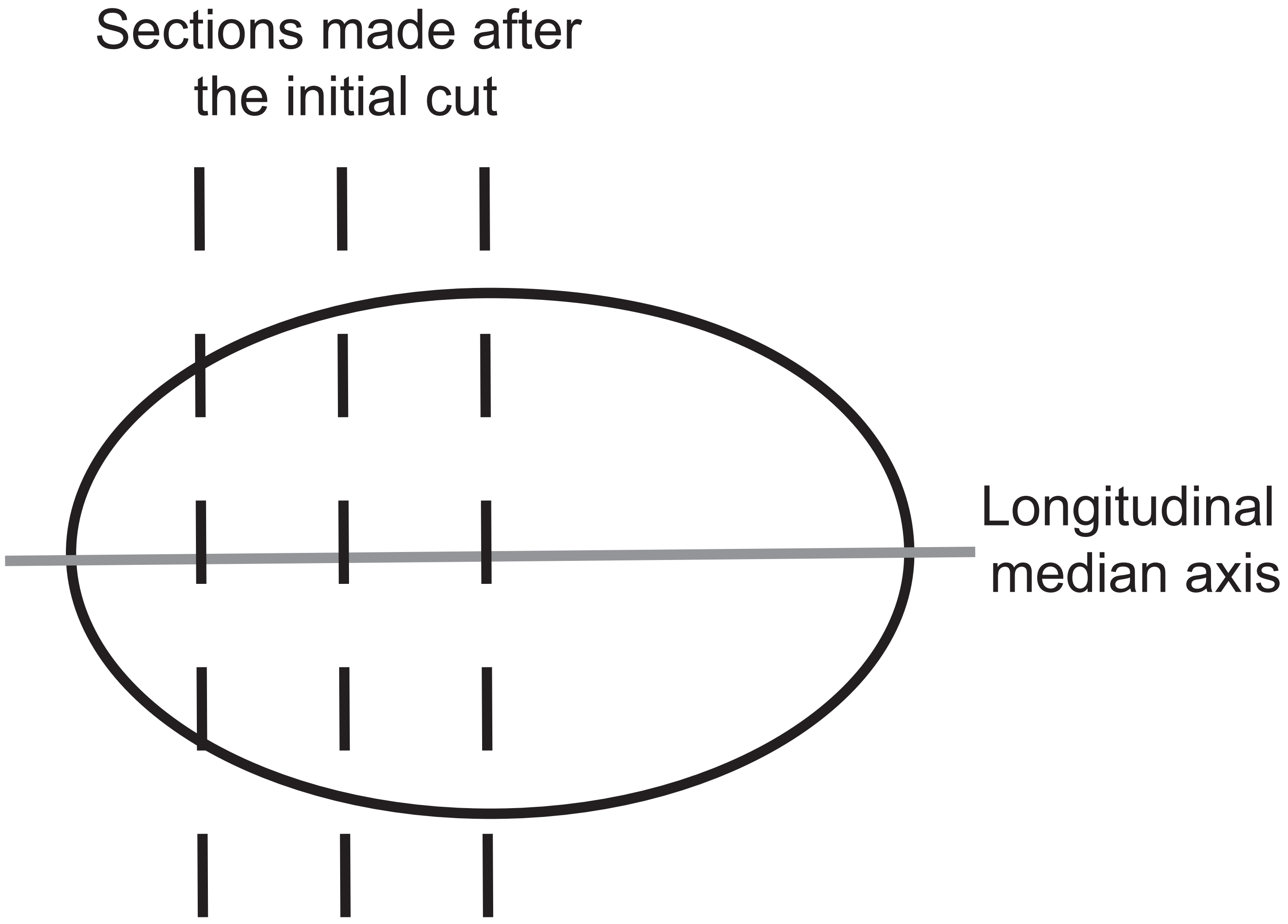

Supplement: Supplementary file 1 — Appendix S1. An illustration of how to slice a coal ball. Slabs (2.5–4 cm thick; represented by dashed black lines) should be cut perpendicular to the coal ball's longitudinal median axis (solid gray line). [file APS3-11-e11556-s001.pdf]
